# Supplementary material for: Modeling glioblastoma heterogeneity as a dynamic network of cell states
Source: Mol Syst Biol. 2021 Sep 16;17(9):e10105. doi: 10.15252/msb.202010105 (PMC8444284; doi:10.15252/msb.202010105)
Supplement: Supplementary file 5 — Source Data for Figure 3 [file MSB-17-e10105-s001.zip › Figure3A_sourcedata/GSEA_3065/hallmarks_state4.GseaPreranked.1623416296068/HALLMARK_UV_RESPONSE_DN.html]

Details for gene set HALLMARK\_UV\_RESPONSE\_DN[GSEA]

|  || Dataset | state4 |
| Phenotype | NoPhenotypeAvailable |
| Upregulated in class | na\_pos |
| GeneSet | HALLMARK\_UV\_RESPONSE\_DN |
| Enrichment Score (ES) | 0.26486102 |
| Normalized Enrichment Score (NES) | 1.0862825 |
| Nominal p-value | 0.29069766 |
| FDR q-value | 0.39075452 |
| FWER p-Value | 0.986 |
Table: GSEA Results Summary

  

Fig 1: Enrichment plot: HALLMARK\_UV\_RESPONSE\_DN      
 Profile of the Running ES Score & Positions of GeneSet Members on the Rank Ordered List

  

| PROBE | GENE SYMBOL | GENE\_TITLE | RANK IN GENE LIST | RANK METRIC SCORE | RUNNING ES | CORE ENRICHMENT || 1 | NFIB |  |  | 75 | 0.269 | 0.0268 | Yes |
| 2 | ID1 |  |  | 84 | 0.261 | 0.0634 | Yes |
| 3 | FYN |  |  | 87 | 0.260 | 0.1007 | Yes |
| 4 | MAP1B |  |  | 111 | 0.243 | 0.1323 | Yes |
| 5 | ANXA2 |  |  | 237 | 0.189 | 0.1395 | Yes |
| 6 | CELF2 |  |  | 324 | 0.170 | 0.1503 | Yes |
| 7 | BHLHE40 |  |  | 451 | 0.143 | 0.1508 | Yes |
| 8 | SRI |  |  | 458 | 0.143 | 0.1705 | Yes |
| 9 | ANXA4 |  |  | 461 | 0.142 | 0.1908 | Yes |
| 10 | COL1A2 |  |  | 466 | 0.142 | 0.2107 | Yes |
| 11 | ATRX |  |  | 725 | 0.111 | 0.1851 | Yes |
| 12 | PMP22 |  |  | 765 | 0.107 | 0.1943 | Yes |
| 13 | IGFBP5 |  |  | 901 | 0.096 | 0.1863 | Yes |
| 14 | COL11A1 |  |  | 905 | 0.095 | 0.1997 | Yes |
| 15 | NR1D2 |  |  | 919 | 0.094 | 0.2112 | Yes |
| 16 | SPOP |  |  | 1078 | 0.083 | 0.1977 | Yes |
| 17 | PRKAR2B |  |  | 1084 | 0.083 | 0.2089 | Yes |
| 18 | ADD3 |  |  | 1118 | 0.081 | 0.2153 | Yes |
| 19 | SIPA1L1 |  |  | 1130 | 0.080 | 0.2252 | Yes |
| 20 | ICA1 |  |  | 1134 | 0.080 | 0.2362 | Yes |
| 21 | DDAH1 |  |  | 1153 | 0.078 | 0.2447 | Yes |
| 22 | AKT3 |  |  | 1190 | 0.077 | 0.2500 | Yes |
| 23 | TFPI |  |  | 1213 | 0.075 | 0.2573 | Yes |
| 24 | CAP2 |  |  | 1276 | 0.072 | 0.2577 | Yes |
| 25 | AGGF1 |  |  | 1315 | 0.070 | 0.2618 | Yes |
| 26 | APBB2 |  |  | 1358 | 0.068 | 0.2649 | Yes |
| 27 | CDKN1B |  |  | 1650 | 0.057 | 0.2261 | No |
| 28 | MAP2K5 |  |  | 1880 | 0.050 | 0.1963 | No |
| 29 | VLDLR |  |  | 1952 | 0.048 | 0.1918 | No |
| 30 | BCKDHB |  |  | 2036 | 0.046 | 0.1851 | No |
| 31 | SYNE1 |  |  | 2057 | 0.045 | 0.1884 | No |
| 32 | PRKCA |  |  | 2232 | 0.041 | 0.1662 | No |
| 33 | NR3C1 |  |  | 2265 | 0.040 | 0.1668 | No |
| 34 | PIK3R3 |  |  | 2273 | 0.040 | 0.1715 | No |
| 35 | ATXN1 |  |  | 2416 | 0.037 | 0.1538 | No |
| 36 | PDLIM5 |  |  | 2517 | 0.034 | 0.1426 | No |
| 37 | COL5A2 |  |  | 2672 | 0.031 | 0.1223 | No |
| 38 | PLPP3 |  |  | 2706 | 0.030 | 0.1214 | No |
| 39 | ACVR2A |  |  | 2753 | 0.030 | 0.1182 | No |
| 40 | ARHGEF9 |  |  | 2996 | 0.025 | 0.0827 | No |
| 41 | F3 |  |  | 3133 | 0.022 | 0.0640 | No |
| 42 | RXRA |  |  | 3145 | 0.022 | 0.0654 | No |
| 43 | PTEN |  |  | 3314 | 0.018 | 0.0408 | No |
| 44 | YTHDC1 |  |  | 3393 | 0.016 | 0.0305 | No |
| 45 | NIPBL |  |  | 3414 | 0.015 | 0.0295 | No |
| 46 | MAGI2 |  |  | 3482 | 0.013 | 0.0205 | No |
| 47 | PHF3 |  |  | 3492 | 0.012 | 0.0208 | No |
| 48 | TJP1 |  |  | 3531 | 0.011 | 0.0163 | No |
| 49 | ERBB2 |  |  | 3828 | -0.000 | -0.0315 | No |
| 50 | TOGARAM1 |  |  | 3913 | -0.004 | -0.0445 | No |
| 51 | NOTCH2 |  |  | 3944 | -0.006 | -0.0484 | No |
| 52 | EFEMP1 |  |  | 4012 | -0.010 | -0.0578 | No |
| 53 | KALRN |  |  | 4089 | -0.015 | -0.0679 | No |
| 54 | GJA1 |  |  | 4123 | -0.017 | -0.0708 | No |
| 55 | MTA1 |  |  | 4269 | -0.027 | -0.0904 | No |
| 56 | ATP2B4 |  |  | 4292 | -0.028 | -0.0898 | No |
| 57 | ATRN |  |  | 4332 | -0.031 | -0.0916 | No |
| 58 | SFMBT1 |  |  | 4353 | -0.032 | -0.0902 | No |
| 59 | FZD2 |  |  | 4375 | -0.034 | -0.0886 | No |
| 60 | RASA2 |  |  | 4409 | -0.036 | -0.0887 | No |
| 61 | IGF1R |  |  | 4432 | -0.038 | -0.0867 | No |
| 62 | HAS2 |  |  | 4502 | -0.045 | -0.0914 | No |
| 63 | ADORA2B |  |  | 4561 | -0.049 | -0.0937 | No |
| 64 | MYC |  |  | 4618 | -0.053 | -0.0951 | No |
| 65 | CDK13 |  |  | 4859 | -0.072 | -0.1234 | No |
| 66 | SLC7A1 |  |  | 4953 | -0.079 | -0.1270 | No |
| 67 | NRP1 |  |  | 4994 | -0.082 | -0.1215 | No |
| 68 | KCNMA1 |  |  | 5231 | -0.104 | -0.1446 | No |
| 69 | DLG1 |  |  | 5592 | -0.144 | -0.1820 | No |
| 70 | ATP2B1 |  |  | 5594 | -0.144 | -0.1613 | No |
| 71 | MMP16 |  |  | 5734 | -0.168 | -0.1594 | No |
| 72 | FHL2 |  |  | 5776 | -0.179 | -0.1401 | No |
| 73 | LAMC1 |  |  | 5795 | -0.182 | -0.1166 | No |
| 74 | SERPINE1 |  |  | 5812 | -0.186 | -0.0922 | No |
| 75 | MET |  |  | 5815 | -0.187 | -0.0655 | No |
| 76 | CAV1 |  |  | 5842 | -0.193 | -0.0416 | No |
| 77 | LDLR |  |  | 5843 | -0.194 | -0.0135 | No |
| 78 | SYNJ2 |  |  | 6073 | -0.279 | -0.0100 | No |
| 79 | CACNA1A |  |  | 6074 | -0.280 | 0.0306 | No |
Table: GSEA details [plain text format]

  

Fig 2: HALLMARK\_UV\_RESPONSE\_DN: Random ES distribution      
 Gene set null distribution of ES for **HALLMARK\_UV\_RESPONSE\_DN**
